# Cardiorenal Syndrome: From Hemodynamic and Humoral Pathophysiology to New Frontiers in Modulation and Enabling Therapy—Part 1

**DOI:** 10.3390/biomedicines14102327

**Published:** 2026-10-13

**Authors:** Alfredo Mauriello, Valeria Cetoretta, Carmen Del Giudice, Adriana Correra, Anna Chiara Maratea, Renato Gioia, Biagio Liccardo, Giovanni Esposito, Vincenzo Russo, Antonello D’Andrea

**Affiliations:** 1 S. C. Cardiology, Institute National Cancer, IRCCS, Fondazione “G. Pascale”, Via M. Semmola 52, 80131 Naples, Italy; alfredo.mauriello93@libero.it; 2 Cardiology and Arrhythmology Clinic, University Hospital “Ospedali Riuniti”, Marche Polytechnic University, Via Conca 71, 60126 Ancona, Italy; vcetoretta@gmail.com; 3 Cardiology Unit, Boscotrecase Hospital, ASL NA3Sud, Via Lenza 3, 80042 Boscotrecase, Italy; carmen.delgiu93@gmail.com; 4 Cardiology Department, Ospedali Riuniti University Hospital, Viale Pinto 1, 71122 Foggia, Italy; adrianacorrera@gmail.com; 5 Department of Cardiovascular Disease, ASL Napoli 1 Centro, Via Comunale del Principe 13a, 80145 Napoli, Italy; annachiara.maratea@gmail.com; 6 Cardiology and Intensive Care Unit, Department of Cardiology, Umberto I Hospital, Via A. de Nicola 1, 84014 Nocera Inferiore, Italy; renatogioia@aslsalerno.it; 7 Cardiology Unit, Department of Medical and Translational Sciences, Monaldi Hospital, University of Campania “Luigi Vanvitelli”, Via L. Bianchi snc, 80131 Naples, Italy; liccardob@gmail.com (B.L.); vincenzo.russo@unicampania.it (V.R.); 8 Cardiology Unit, Department of Advanced Biomedical Sciences, University of Naples Federico II, Via S. Pansini 5, 80131 Naples, Italy; giovanni.esposito2@unina.it

**Keywords:** cardiorenal syndrome, biomarker, venous congestion, oxidative stress

## Abstract

Cardiorenal syndrome (CRS) encompasses a spectrum of disorders in which acute or chronic dysfunction of the heart or the kidney induces dysfunction in the other. Although the five-subtype classification provides a useful conceptual framework, operational definitions remain heterogeneous and management requires an individualized approach. Traditionally attributed to reduced forward arterial perfusion, renal impairment in heart failure is increasingly associated with systemic venous congestion and renal venous hypertension, although most evidence is observational. At the humoral level, chronic activation of the renin–angiotensin–aldosterone system and the sympathetic nervous system sustains a cycle of oxidative stress, inflammation, and fibrosis. In this first part of a narrative review, we summarize these hemodynamic and humoral mechanisms and examine biomarkers that complement serum creatinine, including markers of filtration, tubular injury, and fibro-inflammatory activity, emphasizing that their interpretation depends on baseline renal function and congestion status. PubMed, Scopus, and Web of Science were searched up to September 2026. The available evidence is mainly observational and heterogeneous; these biomarkers appear complementary rather than substitutive, and their incremental clinical value requires prospective validation. Therapeutic strategies will be addressed in Part 2.

## 1. Introduction

Cardiorenal syndrome (CRS) encompasses a spectrum of disorders involving both the heart and kidneys in which acute or chronic dysfunction in one organ may induce acute or chronic dysfunction in the other organ [1]. In this dynamic relationship, heart failure (HF) can trigger or worsen a decline in kidney function, and conversely, renal impairment can severely impact the heart [2]. Renal dysfunction is highly prevalent in HF, affecting approximately 40–50% of patients, and represents a major determinant of adverse outcomes [3]. This mutual decline is driven by intricate neurohormonal feedback mechanisms, where damage in one organ inevitably sparks or accelerates injury in the other [1]. For a long time, the absence of a standardized definition left clinicians navigating significant uncertainty regarding how to diagnose and manage the condition properly. To address this and enable a more tailored clinical approach, a classification system dividing CRS into five distinct subtypes was introduced [4]. Accordingly, in type 1 and 2 CRS, worsening of acute HF (type 1) or chronic HF (type 2) leads to kidney dysfunction. In types 3 and 4 (termed acute and chronic renocardiac syndromes, respectively), consisting of acute kidney injury (AKI) or chronic kidney disease (CKD) causes worsening of HF. In type 5 CRS, various systemic conditions cause simultaneous worsening of the heart and kidney [4]. Type 1 CRS is the most common type (49%), followed by type 2 (23%) and type 4 (20%) [5]. While the classic Ronco classification [4] into five subtypes remains the fundamental framework to define the primary driver and acuity of organ cross-talk, modern clinical practice increasingly views CRS through an integrated cardio-kidney-metabolic (CKM) continuum [6]. This updated perspective acknowledges that rigid subtype boundaries often overlap in chronic multimorbid patients, shifting the focus toward phenotypic profiling based on hemodynamic congestion and systemic neurohormonal/fibro-inflammatory drivers.

This narrative review was not conducted as a systematic review. PubMed, Scopus and Web of Science were searched up to September 2026, without a lower date limit for landmark studies; reference lists of key articles and guidelines were also screened. Search terms combined “cardiorenal syndrome” with “venous congestion,” “worsening renal function,” “oxidative stress,” “renin–angiotensin–aldosterone system,” biomarker terms (“cystatin C,” “NGAL,” “KIM-1,” “NT-proBNP,” “troponin,” “galectin-3,” “sST2,” and “proenkephalin”) and ultrasound indices (“VExUS,” “renal venous stasis index”). We considered original human studies in adults with heart failure or cardiorenal syndrome (randomized trials, prospective and retrospective cohorts, and post hoc analyses), meta-analyses and international guidelines; experimental studies were used for mechanistic questions. Articles were selected by A.M. and discussed with A.D.A.; disagreements were resolved by consensus. No formal quality assessment was performed, so the strength of evidence is discussed in the text where relevant.

The aim of our comprehensive review is to describe the pathophysiological mechanisms of CRS and subsequently outline the diagnostic and prognostic markers of CRS.

## 2. Pathophysiology of Cardiorenal Syndrome

### 2.1. Hemodynamic Mechanisms: Venous Congestion Versus Hypoperfusion

For many years, CRS was mainly interpreted as the result of reduced cardiac output (CO) and impaired renal arterial perfusion. The traditional “forward failure” model remains valid in patients with low-output HF, persistent hypotension, or cardiogenic shock [7]. However, it does not fully explain the kidney dysfunction observed in many patients with preserved blood pressure (BP), adequate cardiac index (CI), or predominant right-sided congestion [3].

Glomerular filtration rate (GFR) is determined by the net ultrafiltration pressure across the glomerular capillary, i.e., glomerular capillary hydrostatic pressure minus hydrostatic pressure in Bowman’s space and plasma oncotic pressure, and is modulated by afferent and efferent arteriolar tone. Renal perfusion pressure depends on both mean arterial pressure and renal venous pressure, which approximates central venous pressure (CVP). When right atrial pressure and CVP rise, the increase is transmitted backward to the renal veins, reducing the transrenal perfusion gradient. Elevated renal venous pressure also raises renal interstitial hydrostatic pressure; because the kidney is enclosed within a poorly distensible capsule, even modest increases may compress peritubular capillaries and tubules and increase intratubular pressure, thereby depends on the balance between arterial inflow pressure and venous outflow pressure [8]. In patients, these effects may be amplified by increased intra-abdominal pressure [9]. Congestion is also accompanied by activation of the renin–angiotensin–aldosterone and sympathetic nervous systems, sodium and water retention, inflammation, and endothelial dysfunction, which are discussed in Section 2.2. The consequence is impaired glomerular filtration, sodium avidity, progressive volume expansion, and reduced responsiveness to diuretic therapy. Thus, the congested kidney is not merely hypoperfused; it is also exposed to increased venous and interstitial pressure and intrarenal edema, which may contribute to renal dysfunction [10].

Renal dysfunction in advanced HF cannot be explained by reduced forward flow alone. In a post hoc analysis of the Evaluation Study of Congestive Heart Failure and Pulmonary Artery Catheterization Effectiveness (ESCAPE) trial [11], Nohria et al. evaluated 433 patients hospitalized with advanced decompensated HF (mean age of 56 ± 14 years; 74% male) randomized to pulmonary artery catheter-guided therapy (PAC, *n* = 215) or clinical assessment-guided therapy (CLIN, *n* = 218), with no relevant baseline differences between groups in age, hypertension, diabetes, serum creatinine or eGFR. In the PAC subgroup, right atrial pressure (RAP) showed a weak but statistically significant correlation with baseline serum creatinine (r = 0.165, *p* = 0.03) and an inverse correlation with baseline eGFR (r = −0.195, *p* = 0.01), whereas cardiac index, pulmonary capillary wedge pressure and systemic vascular resistance were not significantly associated with baseline renal function [12].

Mullens et al. prospectively evaluated 145 consecutive patients (mean age of 57 ± 14 years; 74% male) with advanced decompensated heart failure (ADHF) treated with PAC-guided intensive medical therapy [13]. Worsening renal function (WRF), defined as an increase in serum creatinine ≥ 0.3 mg/dL, occurred in 58 patients (40%). Patients who developed WRF had higher CVP both at admission (18 ± 7 vs. 12 ± 6 mmHg; *p* < 0.001) and after treatment (11 ± 8 vs. 8 ± 5 mmHg; *p* = 0.04). Persistent CVP ≥ 8 mmHg was also associated with a higher incidence of WRF (51% vs. 18%; *p* = 0.01). These findings suggest that venous congestion is associated with the development of WRF, but this study is limited by sample size.

In 2557 cardiovascular patients (mean age 59 ± 15 years; 57% men) undergoing right heart catheterization for several diseases, Damman et al. retrospectively found that higher CVP was independently associated with lower eGFR after adjustment for age, sex and cardiac index (*p* < 0.0001) and also predicted all-cause mortality over a median follow-up of 10.7 years (HR 1.03 per mmHg increase, 95% CI 1.01–1.05; *p* = 0.0032) [14]. Taken together, these data indicate that “backward failure” is not only a marker of advanced HF, but also a potential driver of renal injury, with associated worse clinical outcomes [15].

Venous congestion can promote renal injury in patients with right-sided heart failure, tricuspid regurgitation (TR) and significant volume overload. In a cohort of 1234 patients (median age 72 years [IQR 63–78]; 49.6% men) with moderate-to-severe secondary TR, Butcher et al. retrospectively showed that significant renal impairment, defined as eGFR < 60 mL/min/1.73 m^2^, was present in 45% of patients. Renal dysfunction was more frequent when TR was associated with right ventricular dysfunction, defined by a tricuspid annular plane systolic excursion (TAPSE) below 14 mm (OR 1.49, 95% CI 1.11–1.99; *p* = 0.008). These findings suggest that TR can promote renal impairment through worsening systemic venous congestion and increasing renal venous pressure [16].

The renal venous stasis index (RVSI) is a Doppler-derived parameter calculated as:

RVSI = (cardiac cycle time − venous flow time)/cardiac cycle time.

It represents the proportion of the cardiac cycle during which renal venous outflow is absent; higher values indicate more severe renal venous stasis [17].

In a prospective observational study including 388 patients with advanced HF (median age 71 years (IQR 63.0–79.8), 58% male), Ohara et al. found that higher RVSI was associated with lower eGFR (*p* = 0.017) and higher urinary albumin-to-creatinine ratio (*p* = 0.008). Consistently, RVSI correlated with RAP (R = 0.253), inferior vena cava (IVC) diameter (R = 0.327), and TR severity (R = 0.197; all *p* < 0.001), but not with cardiac index (R = −0.019; *p* = 0.769) or left ventricular function (LVEF) (R = −0.063; *p* = 0.215). High RVSI independently predicted cardiac events (HR 1.908, 95% CI 1.046–3.479; *p* = 0.035). Thus, RVSI identifies right-sided renal venous congestion and worse prognosis in HF patients [18].

Blood redistribution from the splanchnic venous reservoir, elevated intra-abdominal pressure, and volume overload can all raise renal venous pressure and contribute to renal dysfunction, even in the absence of overt weight gain [19]. The Venous Excess Ultrasound score (VExUS) provides a noninvasive tool to evaluate clinically relevant venous congestion and to guide decongestive therapy through the combined assessment of the IVC, hepatic, portal, and intrarenal venous Doppler patterns [20,21]. Higher VExUS grades, therefore, identify a more advanced congestion phenotype, which has been associated with impaired natriuretic and diuretic response, WRF, and adverse in-hospital outcomes [22]. In a prospective, single-center observational study of 100 patients [median age of 73.5 years (64–81), with 60% men] admitted to the intensive cardiac unit (ICU) with acute HF, Sovetova et al. showed that pathological intrarenal venous Doppler patterns, particularly monophasic renal venous flow, have been associated with lower eGFR (*p* < 0.001), higher creatinine at admission and peak creatinine (both *p* < 0.001), WRF (RR 4.31, 95% CI 2.90–6.41; *p* < 0.001), and diuretic resistance (OR 28.38, 95% CI 7.66–105.13; *p* < 0.001) [23]. Furthermore, while the monophasic intrarenal venous Doppler pattern proved to be a powerful isolated predictor of renal impairment and diuretic resistance, a direct correlation with short-term (30-day) mortality in this cohort emerged primarily when this alteration was combined with a severe systemic congestion phenotype, as identified by a VExUS grade 3 score. Serum creatinine alone is an incomplete marker of renal injury during decongestive therapy, because of a transient rise in creatinine during effective diuresis that may reflect hemoconcentration or functional changes in filtration pressure rather than true structural kidney damage. For this reason, changes in renal function should be interpreted together with the degree of decongestion, urine output, natriuresis, blood pressure, and overall clinical response. Thus, venous congestion should not be interpreted simply as a consequence of advanced HF, but rather as a factor that may contribute to renal injury in the cardiorenal syndrome [23].

Importantly, the evidence summarized above supports congestion as a patho-physiological and prognostic marker, but it does not demonstrate that ultra-sound-guided decongestion improves clinical outcomes. A systematic review and me-ta-analysis of VExUS [24], including 565 patients by five studies showed that a VExUS grade ≤ 1 is associated with lower in-hospital mortality among patients hospitalized for AHF (OR: 0.175; 95% CI: 0.061–0.497). In the randomized CAVA-ADHF-DZHK10 trial, including 388 patients (mean age 70 years, 70% male) decongestion guided by daily IVC ultrasound added to clinical assessment did not improve decongestion, measured as NT-proBNP reduction from admission to discharge, compared with clin-ical assessment alone in acute decompensated HF (95% CI: −9.4% to 22.6%; *p* = 0.58) [25]. Prospective trials testing VExUS- or RVSI-guided strategies on clinical endpoints are therefore needed before these indices can be recommended to guide therapy.

### 2.2. Humoral Mechanisms: RAAS Activation, Sympathetic Nervous System, Inflammation, and Oxidative Stress

Oxidative stress (OS) represents a central maladaptive pathway in chronic HF, contributing to the progression of cardiorenal dysfunction. In the early stages of HF, activation of the renin–angiotensin–aldosterone system (RAAS) and the sympathetic nervous system (SNS) acts as a compensatory response to reduced effective arterial perfusion [26]. These mechanisms help preserve blood pressure, CO and organ perfusion through vasoconstriction, increased myocardial contractility, and sodium–water retention. However, when neurohormonal activation persists, this initially adaptive response becomes harmful. Sustained RAAS and SNS stimulation increases preload, afterload and venous congestion, while promoting renal vasoconstriction, sodium retention, inflammation, OS, myocardial and renal fibrosis, adverse ventricular remodeling, and progressive loss of renal function [27].

Angiotensin II (Ang II) is a major mediator of this process. Through AT1 receptor activation, Ang II promotes intrarenal vasoconstriction, tubular sodium reabsorption, inflammatory signaling, and excessive generation of reactive oxygen species (ROS) [28]. In experimental murine models and renal tubular cell cultures, Haugen et al. showed that Ang II increased renal oxidative stress markers and was associated with reduced creatinine clearance and increased proteinuria (both *p* < 0.05) [29]. Persistent RAAS and SNS activation increases ROS production through nicotinamide adenine dinucleotide phosphate oxidase (NADPH oxidase/NOX) activation, mitochondrial dysfunction, and disruption of the nitric oxide (NO)/ROS balance [30]. Excess ROS damages endothelial and tubular cells and activates profibrotic pathways, including transforming growth factor-β1 (TGF-β1) signaling with extracellular matrix deposition and tubulointerstitial fibrosis [31]. Furthermore, RAAS-mediated intrarenal vasoconstriction and hypoperfusion reduce eGFR with retention of uremic toxins, including asymmetric dimethylarginine (ADMA), an endogenous inhibitor of nitric oxide synthase (NOS). By reducing NO bioavailability, ADMA impairs endothelial homeostasis and favors renal vasoconstriction, microvascular dysfunction and progressive renal damage. In parallel, chronic sympathetic overactivity contributes to systemic and renal vasoconstriction, enhances RAAS activation, and promotes sodium and water retention. These effects increase cardiac afterload, aggravate venous congestion and impair renal perfusion [32]. At the myocardial level, persistent adrenergic stimulation promotes fibrosis, adverse ventricular remodeling, arrhythmias and progressive contractile dysfunction. Norepinephrine may also directly stimulate NOX-dependent ROS generation in the heart and vasculature, thereby contributing to OS during chronic sympathetic activation [33].

Several biomarkers have been investigated as indicators of neurohormonal activation and disease severity in HF, including natriuretic peptides, catecholamines, neprilysin, and endothelin-1 [26]. Although OS biomarkers have been explored in HF, their use remains less established for routine clinical assessment. Because ROS are highly unstable and difficult to measure directly, oxidative damage is usually evaluated through indirect biomarkers [34]. Among these, urinary isoprostanes have been associated with RAAS activation and renal dysfunction, supporting a close relationship between OS and neurohormonal activation in chronic heart failure with WRF [35]. The main biomarkers and their pathophysiological relevance are summarized in Table 1.

### 2.3. Inflammation, Resolution, and Immunometabolic Remodeling

Beyond neurohormonal activation and oxidative stress, cardiorenal syndrome is accompanied by a systemic fibro-inflammatory state, which is particularly relevant in patients with obesity and the cardio-kidney-metabolic phenotype [6]. Obesity-associated low-grade inflammation arises from intersecting pathways, including region-specific adipose maladaptation, altered adipokine signaling and gut microbiota disturbances [36]. In HF, congestion-related and metabolic stressors may add to this inflammatory load; however, the contribution of each pathway to renal injury has not been defined.

Inflammation is terminated by active resolution programs, mediated by specialized pro-resolving lipid mediators and by reprogramming of monocytes and macrophages toward a reparative phenotype. Impaired resolution has been proposed as a reason why low-grade inflammation persists and drives fibrosis, and “resolution pharmacology” has been proposed as a strategy complementary to inhibition of pro-inflammatory pathways [36]. Closely related is immunometabolic remodeling, in which changes in cellular metabolism sustain pro-inflammatory and profibrotic immune phenotypes.

Importantly, this evidence is largely mechanistic and derives from experimental models and from obesity-related cardiovascular disease, not from cardiorenal syndrome specifically. Its application to the cardio–renal axis should therefore be regarded as a working hypothesis. Therapeutic implications will be discussed in Part 2.

Thus, disease-modifying therapies for HF may limit OS by reducing maladaptive neurohormonal activation. RAAS inhibition can decrease Ang II-driven ROS generation, improve NO bioavailability and attenuate endothelial dysfunction. Sacubitril/valsartan has also been associated with lower markers of oxidative injury, including NOX2 activity and 8-isoprostane.

Aldosterone overactivity has been associated with vascular inflammation, OS, and extracellular matrix turnover in chronic HF. In an observational study by Kotlyar et al., including 58 patients with chronic, stable HF not treated with aldosterone antagonists (median age 58 years [IQR 26], 67% men), after multivariable adjustment, plasma aldosterone levels were independently associated with 8-iso-PGF2α, a marker of lipid peroxidation (r = 0.55; *p* = 0.003), intercellular adhesion molecule-1 (ICAM-1), a marker of endothelial inflammation (r = 0.56; *p* = 0.008), and tissue inhibitor of metalloproteinases-1 (TIMP-1), a marker of matrix turnover (r = 0.67; *p* = 0.006) [37]. Mineralocorticoid receptor antagonists may counteract this pathway by reducing aldosterone-mediated oxidative and profibrotic activity [34].

Among angiotensin-converting enzyme (ACE) inhibitors, sulfhydryl-containing agents, such as zofenopril, can enhance NO bioavailability and reduce lipid peroxidation. Beta-blockers, especially carvedilol, bisoprolol, metoprolol and nebivolol, may also reduce free radical production and improve endothelial function [38].

In cardiorenal syndrome, sodium/glucose cotransporter 2 inhibitors (SGLT2i) increase natriuresis and distal sodium delivery, restore tubuloglomerular feedback and reduce glomerular hyperfiltration [39]. They also help relieve renal congestion and interstitial pressure. Beyond these hemodynamic effects, SGLT2i reduce proximal tubular oxygen demand and energy workload, limiting tubular hypoxia, mitochondrial stress, oxidative injury and tubulointerstitial fibrosis [40].

Overall, OS emerges as a key mediator of renal injury in chronic HF and a potential target for future therapeutic strategies in cardiorenal syndrome [41].

## 3. Beyond Creatinine: Emerging and Novel Biomarkers

Biomarkers of renal function in HF are best considered in tiers rather than as a list of alternatives. First-line assessment, supported by current guidelines [2,42], consists of creatinine-based eGFR and urinary albumin-to-creatinine ratio (UACR), together with natriuretic peptides for cardiac assessment. Cystatin C is a second-line test, recommended when creatinine-based eGFR is likely to be inaccurate and a more accurate GFR estimate affects clinical decisions, typically as combined creatinine–cystatin C eGFR. Tubular injury markers, proenkephalin, and fibro-inflammatory markers remain adjunctive or investigational: their role is prognostic or mechanistic, and they are not currently recommended for routine use. For each biomarker, interpretation depends on clinical setting (acute vs. chronic), baseline renal function, and congestion status (Table 1).

Because creatinine and eGFR reflect filtration rather than tissue injury, WRF should not be treated as a single phenotype. At least two biologically distinct situations can be distinguished as follows: (i) a functional, hemodynamic decline in filtration during effective decongestion, in which reduced intraglomerular pressure and hemoconcentration may raise creatinine without tubular injury; and (ii) WRF in the setting of persistent congestion (or hypoperfusion), in which renal venous hypertension, intrarenal edema, or ischemia may cause structural tubular damage. These two phenotypes have different clinical meaning and prognosis, and the biomarkers discussed below are interpreted differently in each.

Albuminuria, measured as UACR, is the second pillar of kidney assessment, after serum creatinine. Albuminuria reflects glomerular and endothelial injury and provides renal and cardiovascular risk information not captured by eGFR. UACR can vary during acute illness and hemodynamic changes, so confirmation on repeated testing in a stable state is advisable before classifying a patient.

Cystatin C is a low-molecular-weight protein produced by all nucleated cells, freely filtered by the glomerulus and catabolized in the proximal tubule. It is less affected than creatinine by muscle mass, which is why it is useful in frail, elderly or sarcopenic patients. However, its levels are also influenced by non-GFR determinants, including obesity, smoking, systemic inflammation, thyroid dysfunction and glucocorticoids, so it is not intrinsically superior to creatinine. KDIGO guidelines [42] recommends starting with creatinine-based eGFR and using combined creatinine–cystatin C eGFR when greater accuracy is needed; the 2026 ESC guidelines [2] similarly give a Class IIa recommendation fgrater cystatin C-based eGFR when creatinine-based estimates are likely to be unreliable. In a prospective single-center observational cohort, Campbell et al. enrolled 240 consecutive patients admitted for acute HF (mean age of 63 ± 14 years and 50% men). After adjustment for age, race, sex, comorbidities, and creatinine, the highest cystatin C quartile remained independently associated with mortality (HR 2.00; *p* = 0.04) and death or rehospitalization (HR 1.94; *p* = 0.002), supporting its role as a renal biomarker for risk stratification in acute HF [43].

The Finnish Acute Heart Failure Study (FINN-AKVA) study, a prospective, observational, multicenter cohort evaluated 480 patients hospitalized for acute HF, with a mean age of 74.8 ± 10.4 years, and 50% were men [44]. Cystatin C was independently associated with 12-month mortality (adjusted HR 3.2, 95% CI 2.0–5.3; *p* < 0.0001). Moreover, among patients with normal plasma creatinine, elevated cystatin C identified a subgroup with higher 12-month mortality compared with patients with both normal creatinine and normal cystatin C (40.4% vs. 12.6%; *p* < 0.0001). These findings show that cystatin C carries prognostic information in acute HF; because of its non-renal determinants, this association may partly reflect inflammation or adiposity rather than glomerular filtration alone.

In a prospective observational cohort from the GeneBank study, Dupont et al. evaluated 823 stable chronic HF patients undergoing elective coronary angiography, with a mean age of 66 ± 11 years and 60% men. During the 3-year follow-up, cystatin C independently predicted major adverse cardiovascular events (MACEs), defined as death, myocardial infarction (MI), or stroke, after adjustment for BNP and clinical risk factors (HR 1.20, 95% CI 1.05–1.36; *p* = 0.0085). Cystatin C also correlated with creatinine (r = 0.73) and eGFR (r = −0.62; all *p* < 0.001). In patients with preserved eGFR, cystatin C improved risk prediction beyond creatinine-based eGFR (AUC 0.600 vs. 0.529; *p* = 0.006; net reclassification improvement 22.2%; and *p* < 0.001) [45]. Thus, cystatin C adds prognostic value to creatinine, particularly in patients with “apparently” preserved renal function. These are prognostic findings and do not by themselves establish superiority for GFR estimation.

In addition, neutrophil gelatinase-associated lipocalin (NGAL) and kidney injury molecule-1 (KIM-1) provide information on tubular stress and injury. NGAL is rapidly upregulated in tubular epithelial cells in response to ischemic, inflammatory or toxic injury, whereas KIM-1 is mainly expressed by injured proximal tubular cells. Tubular injury markers have prognostic relevance in chronic HF, even when eGFR is still preserved [46]. Recent evidence from a meta-analysis of 100 studies including 45,428 patients with HF has shown that higher serum cystatin C was associated with all-cause mortality (adjusted pHR 2.04, 95% CI 1.70–2.43; *p* < 0.05) and the composite outcome of mortality and HF hospitalization (adjusted pHR 2.26, 95% CI 1.73–2.96; *p* < 0.05). Serum NGAL was also associated with mortality (adjusted pHR 2.91, 95% CI 1.49–5.67; *p* < 0.05) and the composite outcome (adjusted pHR 4.11, 95% CI 2.69–6.30; *p* < 0.05). Serum and urinary NGAL predicted WRF (adjusted pHR 2.40, 95% CI 1.48–3.90 and pHR 2.01, and 95% CI 1.21–3.35, respectively; both *p* < 0.05), whereas urinary KIM-1 was associated with WRF (adjusted pHR 1.60, 95% CI 1.24–2.07; *p* < 0.05) but not consistently with mortality or hospitalization [47].

An increase in creatinine during aggressive decongestion does not necessarily indicate structural tubular injury. In a post hoc analysis of the multicenter Renal Optimization Strategies Evaluation (ROSE)-AHF trial, Ahmad et al. evaluated 283 patients hospitalized for acute HF treated with high dose of diuretic (median age 70 years [IQR 62–79], 75% men). WRF, defined as a ≥20% decline in eGFR based on cystatin C, occurred in 21.2% of patients but was not associated with increases in urinary tubular injury biomarkers, including NGAL (*p* = 0.21), KIM-1 (*p* = 0.22), or NAG (*p* = 0.46). This inference is limited by the post hoc design, by the single time window of biomarker sampling, and by the absence of histological confirmation [48]. Conversely, in the absence of effective decongestion, tubular injury markers carry independent information. In the Bio-SHiFT study including a prospective longitudinal cohort of 263 ambulatory patients with stable chronic HF, urinary NAG and KIM-1 added prognostic information: rising tubular biomarkers were independently associated with worse outcomes, with adjusted HR 3.7 (95% CI 1.1–12.6; *p* = 0.034) when either NAG or KIM-1 increased and adjusted HR 8.4 (95% CI 2.6–27.9; *p* < 0.001) when both increased. Patients with rise in all three renal biomarkers had the lowest endpoint-free survival (*p* for trend <0.001) and the highest risk of the composite endpoint (adjusted HR 15.0, 95% CI 2.0–111.0; *p* = 0.008) [49]. Thus, creatinine alone may underestimate renal damage, whereas tubular biomarkers can identify a higher-risk kidney phenotype. In a secondary analysis of the multicenter ROSE-AHF trial, Horiuchi et al. evaluated 339 patients (mean age was 70 ± 12 years; 73% were men) hospitalized for acute HF with kidney dysfunction. Higher baseline urinary KIM-1 and NAG were associated with lower spot urinary sodium concentration (KIM-1: −6.1%, 95% CI −8.5 to −3.7; *p* < 0.001; NAG: −5.9%, 95% CI −9.2 to −2.6; and *p* < 0.001) and lower fractional excretion of sodium (KIM-1: −6.1%, 95% CI −8.5 to −3.6; *p* < 0.001; NAG: −5.2%, 95% CI −8.6 to −3.6; and *p* = 0.001) per two-fold biomarker increase. Higher KIM-1 was also associated with lower total urinary sodium excretion over 72 h (−3.6%, 95% CI −6.8 to −0.2; *p* = 0.037), supporting an association among proximal tubular injury, sodium avidity, and impaired natriuretic response in acute HF [50]. In the international multicenter prospective Acute Kidney Injury Neutrophil Gelatinase-Associated Lipocalin Evaluation of Symptomatic heart failure Study (AKINESIS) cohort, Maisel et al. evaluated 927 patients hospitalized for acute HF and treated with intravenous diuretics (mean age was 68.5 years; 62% were men). WRF or renal replacement therapy within 5 days occurred in 72 patients (7.8%). Plasma NGAL was not superior to creatinine for predicting WRF or renal replacement therapy within 5 days, with similar AUCs for peak NGAL, first NGAL, and first creatinine (0.656, 0.647, and 0.652, respectively). In addition, Plasma NGAL did not show clear incremental value over creatinine for adverse in-hospital events (AUC 0.691 vs. 0.686) [51]. Thus, despite its association with outcomes in pooled analyses [48], NGAL did not outperform creatinine for predicting WRF in the largest acute HF cohort [52], and current evidence does not support its routine use in the acute setting. Its levels are also influenced by inflammation, infection, and chronic kidney disease (Table 1), which may partly explain this discrepancy.

N-terminal pro-B-type natriuretic peptide (NT-proBNP) represents a central biomarker for diagnosis and risk stratification in HF, but its value is strongly influenced by kidney function. As eGFR declines, NT-proBNP concentration tends to increase; therefore, higher NT-proBNP cut-offs may be required [52]. In a pooled analysis of 14,831 patients (mean age 72.1 years, 49.7% men) with heart failure with mildly reduced ejection fraction (HFmrEF) or heart failure with preserved ejection fraction (HFpEF) from Irbesartan in Heart Failure with Preserved Ejection Fraction Study (I-PRESERVE), Treatment of Preserved Cardiac Function Heart Failure with an Aldosterone Antagonist Trial (TOPCAT) Americas, Prospective Comparison of ARNI with ARB Global Outcomes in Heart Failure with Preserved Ejection Fraction (PARAGON-HF) and Dapagliflozin Evaluation to Improve the Lives of Patients with Preserved Ejection Fraction Heart Failure (DELIVER), NT-proBNP remained strongly associated with HF hospitalization or cardiovascular death: each doubling in NT-proBNP increased the risk by 37% (HR 1.37, 95% CI 1.34–1.41), with no significant difference across eGFR categories (*p* for interaction = 0.42). NT-proBNP provided prognostic information across the whole spectrum of kidney function [53]. Of note, patients with reduced eGFR reached the same absolute event risk at lower NT-proBNP levels. Thus, renal dysfunction does not reduce the clinical relevance of NT-proBNP; rather, it identifies patients in whom any given NT-proBNP concentration is associated with greater absolute risk.

The role of high-sensitivity cardiac troponin (hs-cTn) in HF has also been investigated. In an individual patient data meta-analysis of 9289 patients (mean age of 66 ± 12 years, 77% men) with chronic HF from 11 cohorts, Aimo et al. showed that hs-cTnT remained independently associated with all-cause mortality (HR 1.48, 95% CI 1.41–1.55; *p* < 0.001), cardiovascular death (HR 1.40, 95% CI 1.33–1.48; *p* < 0.001) and cardiovascular hospitalization (HR 1.42, 95% CI 1.36–1.49; *p* < 0.001), after adjustment for eGFR and NT-proBNP [54]. Thus, hs-cTnT provides prognostic information in chronic HF beyond renal function.

In a post hoc analysis of the same individual patient dataset, Aimo et al. further compared hs-cTnT, NT-proBNP, hs-CRP, and eGFR for risk stratification in chronic HF. Over a median follow-up of 2.4 years, hs-cTnT showed stronger prognostic discrimination than NT-proBNP for all-cause mortality, cardiovascular mortality, and cardiovascular hospitalization (all *p* < 0.001). eGFR added further prognostic information, being independently associated with all-cause mortality (*p* < 0.001) and cardiovascular mortality (*p* < 0.001). The optimal cut-offs for hs-cTnT and NT-proBNP increased as eGFR declined, suggesting that these biomarkers should be interpreted in relation to renal function rather than using a single fixed threshold [55].

Beyond markers of direct eGFR and tubular injury, the cardiorenal syndrome is also characterized by a systemic and myocardial fibro-inflammatory response. In this context, biomarkers such as Galectin-3 (Gal-3) and soluble suppression of tumorigenicity-2 (sST2) emerge not as purely nephrological indices, but rather as indicators of a broader fibro-inflammatory axis with significant renal cross-talk.

Gal-3 is a β-galactoside-binding lectin mainly released by activated macrophages, although it is also expressed by fibroblasts, endothelial cells and epithelial cells under inflammatory and pro-fibrotic conditions. It is involved in macrophage activation, fibroblast proliferation, collagen deposition and extracellular matrix remodeling [56].

In the kidney, Gal-3 is mainly expressed in tubular compartments and increases after renal injury. Experimental models have linked Gal-3 upregulation to ischemia–reperfusion injury, toxic tubular damage, macrophage infiltration, cytokine release, endothelial activation and tissue remodeling [57].

In a prospective multicenter subanalysis of the Red Española de Insuficiencia Cardiaca (REDINSCOR)-II registry, Caravaca Pérez et al. studied 1201 patients hospitalized for acute HF with available Gal-3 values (mean age was 72 ± 12 years, and 57.6% were men). Gal-3 was inversely correlated with eGFR (rho = −0.51; *p* < 0.001) and increased as renal function declined (trend *p* < 0.001). Although Gal-3 was associated with 12-month mortality in the overall cohort (*p* = 0.038), this association was limited to patients with renal dysfunction (eGFR <60 mL/min/1.73 m^2^; *p* = 0.033) and was absent in those with preserved eGFR (*p* = 0.472). Gal-3 should be interpreted in relation to eGFR rather than as an isolated biomarker [58].

In a post hoc analysis of the prospective AKINESIS cohort, Horiuchi et al. evaluated 790 patients (mean age 69 ± 14 years, and 63% men) hospitalized for acute HF. Higher admission Gal-3 (>25.9 ng/mL) was associated with WRF (*p* < 0.001), markers of renal tubular damage (*p* < 0.001), and myocardial injury with elevated hs-TnT (*p* = 0.009). During hospitalization, higher Gal-3 was associated with AKI, renal tubular damage, myocardial injury, renal replacement therapy, inotrope use, and in-hospital mortality (all *p* < 0.05). At 1 year, after multivariable adjustment, Gal-3 remained independently associated only with mortality (*p* < 0.001), suggesting that it primarily identifies acute HF patients with cardiorenal injury and higher risk of death [59].

In acute HFrEF, higher Gal-3 levels have been associated with early cardiorenal deterioration. In a single-center prospective cohort, Ozyildirim et al. enrolled 166 symptomatic HFrEF patients (mean age 64 years, 70.5% men). Type 1 cardiorenal syndrome (CRS) developed in 41 patients, with no significant differences in age or sex compared with those without CRS. Gal-3 levels were higher in patients who developed CRS (*p* < 0.001) and remained independently associated with CRS after multivariable adjustment (OR 3.21, 95% CI 1.74–5.96; *p* = 0.001). Its discriminative performance was moderate (AUC 0.761, 95% CI 0.676–0.847; *p* < 0.001), supporting Gal-3 as a potential marker of early cardiorenal worsening in acute HFrEF [60].

Overall, Gal-3 may help identify early renal involvement within the cardio–renal–metabolic axis, before kidney dysfunction becomes clinically evident through conventional markers. However, its signal should be interpreted as a marker of broader fibro-inflammatory risk rather than isolated renal injury [61].

sST2 is the circulating isoform of the ST2 receptor, released mainly by cardiomyocytes, cardiac fibroblasts, endothelial cells, immune cells in response to myocardial stretch, inflammation and tissue stress. By binding interleukin-33 (IL-33), sST2 acts as a decoy receptor and attenuates the protective IL-33/ST2 ligand (ST2L) pathway, favoring inflammation, fibrosis and adverse remodeling. It is measured in serum or plasma and is primarily used as a prognostic, rather than diagnostic, biomarker in HF. In advanced HFrEF, sST2 appears to be more closely related to mortality risk than to kidney function decline. In a longitudinal analysis of the prospective multicenter REVIVAL cohort, Roehm et al. evaluated 309 patients with repeated eGFR assessment over 2 years (mean age was 59 years and approximately 74% were men). Unlike Gal-3 and NT-proBNP, which were associated with eGFR decline (*p* = 0.02 and *p* < 0.001, respectively), sST2 showed only a borderline association with renal function decline after full adjustment (*p* = 0.05). Conversely, after accounting for changes in eGFR, sST2 remained independently associated with mortality (*p* = 0.02). These findings suggest that sST2 should be interpreted less as a marker of renal injury progression and more as a prognostic biomarker reflecting a high-risk cardiovascular phenotype in advanced HF [62]. Consistently, in a systematic review and meta-analysis of 10 studies including 1456 patients on chronic hemodialysis, Zhang et al. found that higher sST2 levels were associated with cardiovascular events. The pooled mean age was not reported, while 842 patients (57.8%) were men. Elevated sST2 was associated with an almost three-fold higher risk of cardiovascular events (OR 2.89, 95% CI 1.71–4.89; *p* < 0.0001). These findings support the prognostic relevance of sST2 even in patients with severe renal dysfunction, although the evidence should be interpreted cautiously because most included studies were retrospective and the quality of evidence was limited [63].

Proenkephalin 119–159 (PENK119–159) is an inactive and stable peptide fragment of the endogenous enkephalin system, commonly used as a surrogate biomarker for the activity of biologically active enkephalin peptides. It is freely filtered at the glomerular level, is not protein-bound, and has no relevant tubular or extrarenal elimination. As a result, plasma PENK rises when glomerular filtration declines, reflecting renal function rather than tubular injury [64]. In HF, higher PENK levels have been associated with renal dysfunction and worse clinical outcomes. In a systematic review and meta-analysis of six observational cohort studies, Siranart et al. included 6929 patients (mean age ranged from 63.7 to 76.4 years; the proportion of men ranged from 48.5% to 97.5%) with acute or chronic HF. Elevated PENK was associated with all-cause mortality (pooled HR 1.72, 95% CI 1.62–1.84), HF rehospitalization (pooled HR 1.51, 95% CI 1.38–1.65), and worsening renal function (pooled OR 1.57, 95% CI 1.36–1.82). These findings support PENK as a potential cardiorenal risk marker in HF, although the evidence remains observational and its incremental value over established renal biomarkers requires further validation [65].

In a prospective single-center study of 107 patients presenting to the emergency department with acute HF (mean age 72 ± 13 years; 58% male), point-of-care PENK measured at admission predicted WRF within 48 h after adjustment for systolic blood pressure, eGFR and NT-proBNP levels (adjusted OR 15.4, 95% CI 2.0–120.2; *p* = 0.009), showing moderate discriminative accuracy (AUC 0.690, 95% CI 0.584–0.795; *p* < 0.001). After multivariable analysis, PENK remained independently associated with 90-day mortality (adjusted HR 7.3, 95% CI 1.5–35.0; *p* = 0.013) and 1-year mortality (adjusted HR 7.2, 95% CI 1.7–30.8; *p* = 0.008), whereas its association with 30-day mortality was borderline (*p* = 0.051). However, the single-center design, limited sample size and wide confidence intervals suggest that these findings should be interpreted with caution. Overall, these data support the potential role of PENK as an early adjunctive biomarker for cardiorenal risk stratification in acute HF, particularly for identifying patients at risk of early renal deterioration [66].

The prognostic relevance of PENK in acute cardiorenal risk has also been evaluated beyond HF. In the KID-ACS study, a prospective multicenter derivation–validation study including 7075 patients with acute coronary syndrome (ACS), PENK measured at presentation independently predicted in-hospital AKI (adjusted OR per doubling 1.53; *p* = 0.007) and 30-day mortality (adjusted HR 2.73; *p* < 0.001). Its integration into the six-item KID-ACS score supported combined renal and mortality risk stratification, with AUCs of 0.72 for AKI and 0.91 for 30-day mortality in the derivation cohort. Because these data derive from patients with ACS rather than heart failure, they should be considered hypothesis-generating for cardiorenal risk stratification in HF [67].

WRF during effective decongestion is often functional and prognostically benign, whereas WRF with persistent congestion may signal genuine renal injury; its interpretation should therefore always take congestion status into account, particularly given the heterogeneous definitions used across studies.

Overall, these biomarkers add clinically relevant and prognostic information to the assessment of renal dysfunction in HF, but their current role remains mainly complementary, and larger prospective studies are needed to define their integration into routine risk stratification.

## 4. Limitations

This review has several limitations. First, it is a narrative rather than a systematic review; despite the search strategy described, selection bias cannot be excluded, and no formal quality assessment or meta-analysis was performed. Second, most of the evidence discussed is observational, often post hoc and single-center, which limits causal inference; associations between venous congestion and renal dysfunction may be bidirectional and affected by reverse causation and residual confounding. Third, studies differ in populations (decompensated versus chronic HF, reduced versus preserved ejection fraction), definitions of WRF, biomarker assays, and cut-offs, precluding direct comparison. Fourth, WRF definitions (creatinine- vs. cystatin C-based, different thresholds and time windows) differ across studies, and no study has directly compared biomarker profiles of functional versus structural WRF within the same cohort. Fifth, data on oxidative stress derive largely from experimental and small observational studies. Finally, this first part does not address therapeutic strategies, which will be discussed in Part 2, and renocardiac subtypes (types 3–5) are only marginally covered.

## 5. Conclusions

Venous congestion and neurohormonal activation are central, interrelated mechanisms in cardiorenal syndrome. Available data suggest that congestion contributes to renal dysfunction in heart failure, although causality remains to be established. Serum creatinine remains the reference marker but has important limitations, particularly during decongestion, when its rise may be functional. Cystatin C, tubular injury markers, natriuretic peptides, troponin, galectin-3, sST2, and proenkephalin provide complementary prognostic information, and their interpretation requires knowledge of baseline eGFR and congestion status. Ultrasound indices such as RVSI and VExUS may help characterize congestion, but evidence that guiding therapy with them improves outcomes is lacking. Prospective validation of the incremental value of emerging biomarkers, standardized definitions of worsening renal function, and trials of congestion- or biomarker-guided strategies are needed. Therapeutic approaches will be discussed in Part 2.

## Figures and Tables

**Table 1:** Traditional and emerging biomarkers in cardiorenal syndrome.

Group	Biomarker	Biological Domain	Setting	Role in Cardiorenal Syndrome	Availability	Main Limitation	Supported by Guidelines	Incremental Value
Traditional	Creatinine/eGFR	Filtration	Acute and chronic	Standard assessment of renal function; may worsen during effective decongestion without tubular injury.	High	Late and influenced by muscle mass and volume status.	ESC KDIGO	Reference marker
Traditional	BUN	Neuro-hormonal activation	Acute	Associated with congestion, hypoperfusion and adverse outcomes in HF.	High	Affected by diet, catabolism, bleeding and hydration.	None (observational studies)	Unknown
Traditional	UACR	Glomerular/tubular damage	Chronic	Adds renal and cardiovascular risk information beyond eGFR.	High	Variable during acute illness and hemodynamic changes.	ESC KDIGO	Reference marker
Emerging renal	Cystatin C	Filtration	Acute and chronic	More sensitive filtration marker and independent predictor of HF outcomes.	Intermediate	Affected by inflammation, steroids and thyroid disease.	ESC KDIGO	Modest
Emerging renal	NGAL	Tubular damage	Acute	May identify structural tubular injury; not superior to creatinine in AKINESIS.	Low	Influenced by inflammation, infection and CKD.	None (Prospective cohort; meta-analysis of observational studies)	Not demonstrated
Emerging renal	KIM-1	Tubular damage	Chronic	Associated mainly with worsening renal function; limited outcome evidence.	Low	Urinary assay and non-standardized cut-offs.	None (Prospective cohort; post hoc RCT analysis)	Unknown
Emerging renal	PENK	Filtration	Acute and chronic	Associated with worsening renal function, HF rehospitalization and mortality.	Low	Limited availability and uncertain clinical cut-offs.	None (Meta-analysis of observational studies; single-center cohort)	Unknown
Cardiac/cardiorenal	NT-proBNP	Congestion	Acute and chronic	Prognostic value remains preserved across renal-function categories.	High	Levels rise as eGFR declines.	None (Guideline-endorsed HF; pooled post hoc analysis of RCT)s	Reference marker
Cardiac/cardiorenal	hs-cTnT/hs-cTnI	Myocardial damage	Acute and chronic	Predicts mortality and HF hospitalization independently of eGFR and NT-proBNP.	High	Frequently elevated in CKD without acute coronary syndrome.	None (Individual patient data meta-analysis)	Demonstrated (prognosis)
Cardiac/cardiorenal	Galectin-3	Inflammation and fibrosis	Chronic	Associated with renal dysfunction and adverse remodelling.	Low	Strongly influenced by eGFR; limited incremental value.	None (Registry analysis; post hoc cohort; single-center cohort)	Not demonstrated
Cardiac/cardiorenal	sST2	Inflammation and fibrosis	Chronic	Mainly a prognostic marker of mortality and remodelling.	Low	Not a direct marker of renal injury.	None (Registry analysis; post hoc cohort; single-center cohort)	Unknown

BUN: blood urea nitrogen; CKD: chronic kidney disease; eGFR: estimated glomerular filtration rate; HF: heart failure; hs-cTnI: high-sensitivity cardiac troponin I; hs-cTnT: high-sensitivity cardiac troponin T; KIM-1: kidney injury molecule-1; NGAL: neutrophil gelatinase-associated lipocalin; NT-proBNP: N-terminal pro-B-type natriuretic peptide; PENK: proenkephalin; sST2: soluble suppression of tumorigenicity 2; UACR: urinary albumin-to-creatinine ratio.

## Data Availability

No new data were created or analyzed in this study. Data sharing is not applicable to this article.
